# From pungency perception to consumer preference: The driving role of alkylamide compounds in *Zanthoxylum bungeanum*

**DOI:** 10.1016/j.fochx.2026.103972

**Published:** 2026-05-11

**Authors:** Wen Qiao, Kui Zhong, Bolin Shi, Houyin Wang, Wenxin Wang, Yake Xiang, Lihan Zhang, Kai Su, Na Wang, Lei Zhao

**Affiliations:** a Agriculture and Biotechnology Standardization Institute, China National Institute of Standardization, Beijing 102200, China; b Key Laboratory of Food Sensory Analysis, State Administration for Market Regulation, Beijing 102200, China; c School of Life Science and Engineering, Southwest Jiaotong University, Chengdu 610031, China

**Keywords:** *Zanthoxylum Bungeanum*, Pungency sensation, Consumer preference, Emotional response, Alkylamides

## Abstract

*Zanthoxylum bungeanum* (also known as huajiao) from different origins and varieties exhibits differences in alkylamide content and composition, leading to significant variations in pungency perception and consumer experience. Many studies have reported alkylamide compositions and their effects on pungency sensation, but the specific relationships between alkylamide compounds and consumer experiences remain unclear. The alkylamide composition and consumer responses of 17 huajiao samples were investigated in this study. Compared with red huajiao samples, green huajiao samples had stronger consumer preference. Differences in alkylamide concentration were strongly associated with variations in consumer hedonic responses, and higher percentages of HαSS and sanshool oil among alkylamide compounds were significantly correlated with increased consumer preference. Additionally, huajiao samples with high liking scores were strongly correlated with positive emotions with higher arousal (e.g., active, joyful, and interested). This study provides valuable information on the sensory perception and consumer preferences of huajiao, which can be used for further development and commercialization.

## Highlights

• Liking scores correlate with HαSS and sanshool oil content, but low with HγSS/HγISS.
• Green huajiao elicits high-arousal positive emotions while red evokes calmer positive emotions.
• Preferred samples show stronger trigeminal sensations (tingling, numbing) with reduced bitterness/astringency.

## 1 Introduction

*Zanthoxylum bungeanum* (also known as huajiao) is a traditional spicy ingredient in Chinese cuisine that produces a distinctive numbing and tingling sensation (Liang et al., 2024). It is valued for its significant culinary influence and potential health benefits, and is favored by many consumers worldwide (Sun et al., 2022). The most popular dishes among consumers include spicy hotpot, Mapo tofu, and Sichuan pepper chicken. Additionally, there are many huajiao-flavored snacks and desserts on the market, including tarte Tatin and ice cream (Yue et al., 2019). China is the largest producer of huajiao with an annual output exceeding 500,000 tons, and this is exported to countries and regions including Japan, South Korea, Thailand, the United States, and Europe. Huajiao can be divided into red huajiao (*Zanthoxylum bungeanum* Maxim) and green huajiao (*Zanthoxylum schinifolium* Sieb. et Zucc) based on the color of the peels (Wang et al., 2025), which produce different pungency sensations due to varied pungent chemical compositions (Ni et al., 2022). In China, red huajiao is mainly cultivated in Shaanxi, Sichuan and Gansu provinces, with representative varieties such as Hancheng Dahongpao and Hanyuan nanjiao, while green huajiao is mainly cultivated in Sichuan province and Chongqing municipality, with varieties such as Jinyang and Jiangjin huajiao (Bao et al., 2023).

This pungency sensation primarily comes from the alkylamide compounds in huajiao pericarp, which include multiple alkylamide components, such as hydroxy-α-sanshool (HαSS), hydroxy-β-sanshool (HβSS), hydroxy-γ-sanshool (HγSS), hydroxy-ɛ-sanshool (HɛSS), and others. The alkylamide components have different pungency intensities, For example, HαSS exhibits a stronger pungency intensity than HβSS (Feng et al., 2022). The growing conditions of climate, soil and rainfall significantly affect the growth and development of plants (Zheng et al., 2022), leading to marked differences in the alkylamide compositions and pungency attributes of huajiao from different geographical origins (Zheng et al., 2022). The same results were obtained for the pungency intensity between huajiao oil samples from red and green huajiao material from different production areas (Song, Xia, & Zhong, 2025;). In addition, differences in alkylamide content and composition also directly affected the attributes of pungency perception. Sugai et al. (2005) classified the sensory attributes of pungency into three categories: burning, tingling and numbing, while Zhang et al. (Zhang et al., 2018) defined the sensory attributes of pungency using seven descriptors: burning, numbing, tingling, vibrating, astringency, salivating, and bitter. Both red and green huajiao are widely used in traditional Chinese cooking, but green huajiao is favored by consumers for its unique pungency sensation and aroma (Gao et al., 2024). However, the obvious bitterness of green huajiao sometimes leads to dissatisfaction among consumers (Wang et al., 2025).

The flavor of food is one of the most important factors influencing the sensory quality of food products. It directly determines consumers' preferences, perceptual experiences, and purchasing decisions, thereby contributing to the market acceptance of food products (Bagnulo et al., 2024). Recent studies have shown that food flavor can also evoke positive or negative emotional responses in consumers (Ng et al., 2013). Certain flavors have become central in mental health research, particularly for alleviating negative emotions such as anxiety and depression. For example, sweet taste has been reported to increase agreeableness, while bitter taste increases hostility (Lee & Hong, 2025a). Both salty and sour tastes can lead to significant disgust and surprise reactions, while spicy foods reduce stress by stimulating the release of endorphins (Choy et al., 2021). Huajiao extracts, including hydroxyl-α-sanshool, were found to play a key role in relieving anxiety and reducing cognitive impairments in mouse models (Choudhury et al., 2022; Zhang et al., 2019). These extracts also improved aspects of cognitive performance in a double-blind study of eighty-two healthy human subjects (Kennedy et al., 2019). Similarly, emotional responses are directly related to consumer acceptance and sensory perception. It was reported that people tend to choose foods with either intense, stimulating flavors or soft flavors and textures in response to stress. Intense flavors and crispy or chewy textures trigger high-arousal positive emotions and vivid imagery, while soft flavors and textures induce low-arousal positive emotions (Lee & Hong, 2025b). Additionally, induced stress significantly increases cravings for fattiness, spiciness, and crispness, confirming that emotional stress can stimulate cravings for specific food sensory attributes and influence food choice. Investigating the emotional impact of food flavor on consumers therefore has positive implications for enhancing consumer preferences and improving the ability to predict food choice behavior.

The different varieties of huajiao display varied pungent characteristics due to differences in the composition and concentration of alkylamide compounds, which directly influence consumer preference (Chen et al., 2022). The pungency of Sichuan huajiao positively affects emotional states by regulating neurotransmitters (Wang et al., 2023); further research indicates that this positive emotion is elicited by its pleasant pungent sensation (Liang et al., 2024). These findings increase awareness and interest in the emotional responses evoked by a particular food flavor. However, few studies have examined the differences in consumer preference and emotional responses induced by the sensory attributes of different huajiao samples, particularly regarding the contribution of the concentrations and compositions of pungent compounds to these observed differences.

In particular, the aim of this study is to (1) describe the differences in alkylamide compounds among huajiao samples of different origins and varieties, (2) investigate preference differences between huajiao samples, and (3) explore the correlations between alkylamide compounds and consumer experiences, including preference, sensory perception, and emotions.

## 2 Materials and methods

### 2.1 Materials and reagents

**Fig. 1:**
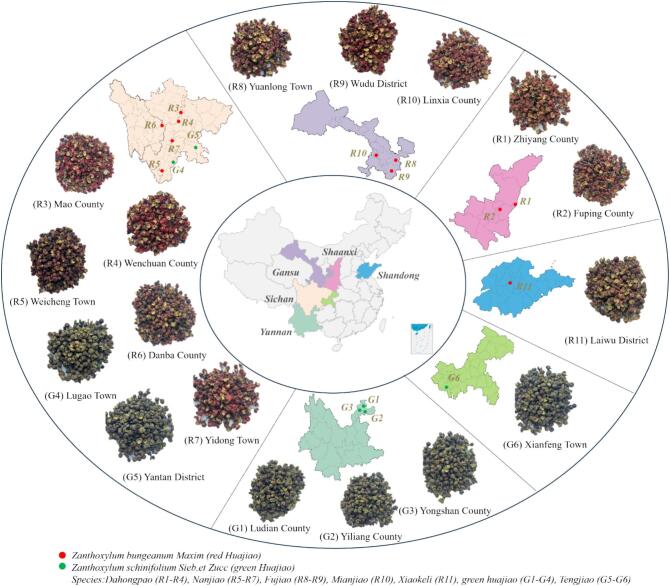
Regional information of 17 huajiao samples.

A total of 17 different dried huajiao materials were purchased from Lihe Flavor (Qingdao) Food Industry Co., Ltd., including 11 red huajiao samples and 6 green huajiao samples. These 17 huajiao samples originated from various major production regions in China and were harvested and sun-dried in August and September 2023. Additionally, these samples represent the main huajiao cultivated species in China. Detailed information, including the variety and cultivation regions, is provided in Fig. 1. Chromatography-grade methanol and acetonitrile were purchased from Fisher Scientific Ltd. (Shanghai, China), while chromatography-grade ethanol and acetic acid were purchased from Henan Rongteng Food Additive Co., Ltd. (Henan, China). Hydroxy-α/β/γ/ε-sanshool (≥98.0%) was sourced from Madsen Technology Co., Ltd. (Chengdu City, Sichuan Province, China). Food-grade ethanol was obtained from Yuyao Longxin Edible Alcohol Co, Ltd. (Yuyao, Zhejiang Province, China). Carboxymethyl cellulose (CMC), Soda crackers, tomato sauce, chopped chili sauce and Jingtian water were sourced from the local supermarket.

### 2.2 Preparation of alkylamide compound extracts

The extraction of alkylamide compounds from huajiao samples was performed according to the method of Chen et al. with minor modifications (Chen et al., 2020). The huajiao particles were crushed twice and then sieved using a 40-mesh sieve. An accurately weighed 5.00 g (±0.01 g) of the processed huajiao powder was transferred to a 250 mL brown glass-stoppered conical flask. The sequential extraction processes included: (1) primary extraction with 75 mL food-grade ethanol under sonication (40 kHz, <40 °C, 20 min); (2) centrifugation (450 ×*g*, 5 min) with collection of the supernatant in a 250 mL brown volumetric flask; (3) secondary extraction of the residue with 25 mL ethanol and collection of the supernatant under the same extraction and centrifugation conditions; and (4) repeating step in (3) and combining the supernatants. The combined supernatants from the three times extractions were hen made up to 250 mL with ethanol to obtain the working solution.

### 2.3 HPLC analysis

A 0.5 mL extract of the pungent substances was placed in a 10 mL brown volumetric flask and filled up with methanol. Then, 1 mL of the solution was withdrawn, and filtered through a 0.22 μm organic filter membrane and transferred to the injection vial for testing. The amide substances were determined using the HPLC method according to the instructions of (Gao et al., 2024). Chromatographic analysis was performed using an Agilent–1200 system (Agilent Technologies Inc., Palo Alto, California, USA) equipped with a ZORBAX-300 SB-C18 column (250 mm × 4.6 mm × 5 μm) Agilent Technologies, Palo Alto, California, USA), using 1% aqueous acetic acid solution (eluent A) and acetonitrile (eluent B) at a flow rate of 0.85 mL/min. The detection wavelength was 270 nm, and the injection volume was 10 μL. The gradient elution conditions for liquid chromatography were as follows: 0–15 min, 60% A, and 40% B; 15.01–40 min, 50% A, and 50% B. Each sample was analyzed in triplicate.

Four alkylamide standards (HαSS, HβSS, HγSS, and HεSS) were accurately weighed and dissolved in methanol to prepare a mixed standard solution. Mixed standard solutions of different concentrations were prepared by further dilution with methanol. The solutions were filtered through a 0.22 μm organic filter membrane before testing. Standard curves for the alkylamide compounds were established, and the correlation coefficient (R^2^) was all above 0.9992. The regression equations for the four alkylamide substances are shown in Supplementary Table 2. Other alkylamide substances were quantified using the external standard method and the standard curve of HβSS.

### 2.4 Consumer test

#### 2.4.1 Participants

Recruit participants in accordance with ISO 11136:2014. Participants were recruited using questionnaires that collected basic information, health status and eating habits. Respondents were asked to describe their acceptance of huajiao-induced pungency sensations. Inclusion criteria required no history of severe allergies, a certain level of acceptance for spicy foods, no anxiety, stress, or recent emotional fluctuations, and participants were instructed not to eat anything within 2 h before the experiment. Finally, 60 consumers were selected (male: female = 22:38, aged 18–28). All participants signed an informed consent form and received cash compensation after the study. This study was approved by the Ethics Committee of Beijing Forestry University, approval number 20220314.

#### 2.4.2 Preparation of huajiao pungency test samples

The results of the pre-test showed that most consumers do not accept the extract in the form of an aqueous solution as a test sample in a consumer test, as there is a significant difference between the tested solution and real pungent food. In daily culinary practice, huajiao is primarily used as an auxiliary seasoning within complex food matrices (Schneider et al., 2014) rather than being consumed alone as a main ingredient. By using a gel-like food as the delivery vehicle, we simulated the carrier-flavor relationship found in real-world diets. This approach aligned the test samples with familiar eating habits, which not only improved consumer acceptance but also ensured that preference and emotional data were collected in a context closer to natural consumption, thereby increasing the ecological validity of the study. Therefore, in this study, we prepared the test samples in the form of gel-like foods.

Weigh 6.00 g of carboxymethyl cellulose (CMC) and add it to 250 mL of boiled water. Stir quickly until the CMC has completely dissolved, ensuring that no visible precipitates or lumps remain. Once dissolved, remove from the heat and add 1.50 g of tomato ketchup and 0.30 g of chili sauce. Mix thoroughly, then filter to remove any solid particles from the chopped chilies. Finally, add 6.0 mL of huajiao extract to the prepared matrix and stir until a homogeneous mass is obtained. Add 10 mL of the mixture to each tasting cup, then place them in the refrigerator at 4 °C for about 5 h to solidify and store until use (no longer than 24 h). Remove the samples and leave them at room temperature for 2 h to allow the temperature to equilibrate before the formal test. We conducted a paired comparison test with *n* = 30 participants. The results showed that 18 assessors indicated that gel-based sample has higher pungent intensity than the water-based sample. According to the one-sided paired comparison table for *n* = 30 and α = 0.05, at least 20 assessors would need to report a difference in pungency between the two samples for the result to be considered statistically significant. Since 18 < 20, the pungency intensity of the matrix sample is not significantly different from that of the gel-based matrix sample (*p* > 0.05), indicating that the matrix does not induce a pungency association in participants.

#### 2.4.3 Sensory perception test

The pungent sensation has seven attributes, including numbing, tingling, vibrating, salivating, bitter, astringency, and burning. The definition of each attribute is consistent with Zhang et al. (Zhang et al., 2018). Each participant rated the intensity of the seven attributes of the test samples using the Rate-All-That-Apply (RATA) method. The advantage of RATA is that it provides better sample discrimination and configuration stability (Ares et al., 2014). The samples were evaluated using a 5-point intensity RATA scale anchored at “very weak”, “weak”, “medium”, “somewhat high” and “high”. The evaluation order of the seven attributes was randomized for each participant to minimize position bias (Pineau et al., 2012). All samples were presented with random three-digit codes and in randomized order. Participants were instructed to refrain from consuming spicy foods for at least 2 h before the experiment and to avoid drinking any liquids other than water for 30 min prior to the assessment. First, consumers were familiarized with the definition of each attribute. Then, they took 2 mL sample with a small spoon, placed it in a cup, put it on the tongue, without chewing, held it for 30 s and then spat it out. After tasting each sample, participants answered the corresponding questions using the APPsense software. Intervals of 5 to 7 min were set between samples, during which participants could cleanse their taste buds with mineral water or biscuits. Evaluation of the next sample was performed only after the numbness had completely subsided.

#### 2.4.4 Hedonic test and emotion test

Hedonic test and emotion test with 17 pungent sensation test samples were carried out in two sessions (7 or 8 samples per session) on two consecutive days. The test conditions complied with the requirements of the ISO 11136:2014 standard. Participants tasted each test sample individually and rated how much they liked the sample using a nine-point hedonic scale (1 = Dislike extremely, 5 = Neither like nor dislike, 9 = Like extremely). Participants then completed a Check-All-That-Apply (CATA) question that included the 25 emotional terms from the EsSense Profile (Nestrud et al., 2016). The question was, “How do you feel after eating this product?” and participants selected all terms that applied (Jaeger et al., 2018). The order of the samples and emotional terms was balanced using a William's Latin Square design (Wakeling & Macfie, 1995). Participants were instructed to rinse their palate with drinking water or eat unsalted soda crackers between two tastings, followed by a 5 to 7 min waiting period to remove the residual pungent sensation. The recovery of oral perception could be confirmed by re-scoring the reference sample.

### 2.5 Statistical analysis

Excel (2019), Origin (2021), and XLSTAT (2019) were used for processing the raw data, graphical representation and ANOVA. A significance level of 5% was used for all data analyses. Principal Component Analysis (PCA) was used to determine differences in the composition of the alkylamide substances. The overall liking scores were analyzed using Agglomerative Hierarchical Clustering (AHC). Cochran's Q test and Correspondence Analysis (CA) were used to identify frequency differences and associations among 25 emotional terms in the test samples. Multiple Factor Intergroup Correlation Heatmap (https://www.chiplot.online/), External Preference Mapping and Multiple Factor Analysis (MFA) were performed using XLSTAT (2019) to assess correlations among preference, sensory perception and emotions.

## 3 Results

### 3.1 Alkylamide coumpounds analysis

**Fig. 2:**
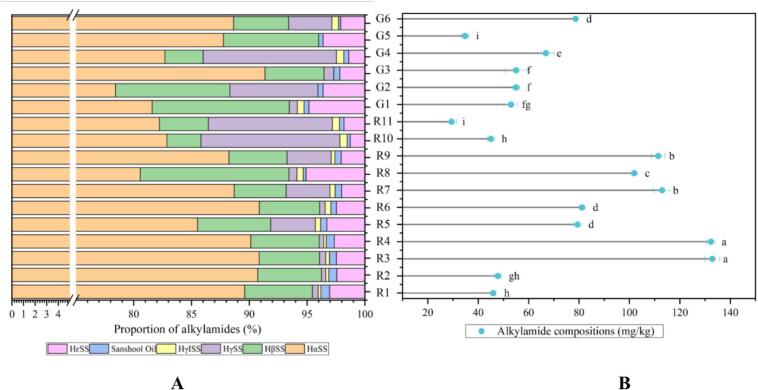
Stacked bar chart of the percentage composition of the 6 alkylamide substances (A) and lollipop of the total alkylamide content (B) for 17 huajiao test samples.

Fig. 2B shows that the total alkylamide content in 17 huajiao samples varied significantly, ranging from 29.27 mg/kg (R11) to 133.08 mg/kg (R3) (*p* < 0.05). Most red huajiao samples had higher total alkylamide content than green huajiao samples. Samples from Sichuan province (R3-R7) and Gansu province (R8-R9) had higher alkylamide concentrations, while samples from Shandong province (R11) and Shaanxi province (R-R2) had lower concentrations. The alkylamide content of most huajiao samples showed great consistency in regional distribution. Six alkylamide compounds were measured in these 17 huajiao samples (Fig. 2A). HαSS was the predominant alkylamide compound, with contents ranging from 78.43% (R10) to 91.35% (G5) of the total alkylamide content. This was followed by HβSS, with contents ranging from 2.93% (R7) to 12.85% (R1). In addition, HγSS and HεSS contents ranged from 0% to 12.03% and 1.27% to 5.09%, respectively. HγISS and sanshool oil had lower contents, ranging from 0% to 0.63% and 0.20% to 0.76%, respectively.

**Fig. 3:**
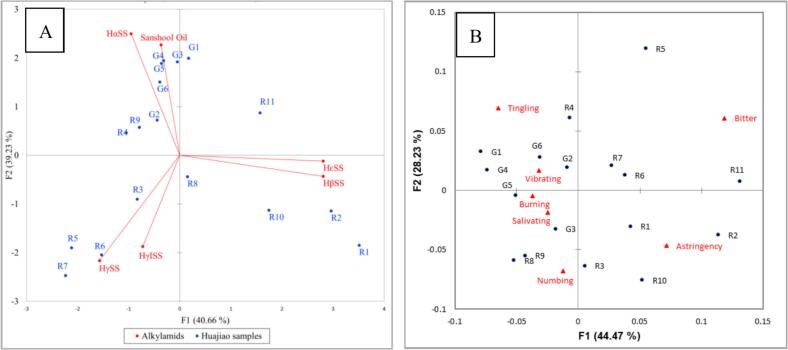
A bi-plot of the first two dimensions generated by Principal Component Analysis of the percentage composition of the 6 alkylamide substances for 17 huajiao samples (blue color for the huajiao samples, red color for the alkylamide substances). B bi-plot of the first two dimensions generated by Correspondence Analysis of 17 huajiao samples (black color for the huajiao samples red color for the Pungency perception). (For interpretation of the references to color in this figure legend, the reader is referred to the web version of this article.)

Fig. 3A shows the PCA biplot of the content of six alkylamide percentage composition and 17 huajiao samples. The first two principal components, F1 (40.66%) and F2 (39.23%), together accounted for 79.89% of the total variance. The six alkylamide compounds were located in three different areas, HαSS and sanshool oil were located in the upper area of the *X* axis and were closely associated with all green huajiao samples and red huajiao samples R4 and R9; HβSS and HεSS were located in the lower right area of the *X* axis and correlated strongly with R1, R2, and R10; HγISS and HγSS were located in the lower left area of the *X* axis and correlated strongly with R3, and R5-R8.

### 3.2 Pungency perception analysis

RATA was used to measure consumers' perception of pungency in this study. The perception results for seven pungency attributes across 17 huajiao samples are shown in Fig. 3B. The first two principal components accounted for 72.70% of the total variance. Most pungency attributes are concentrated on the left side of the *Y* axis, including numbing, tingling, vibrating, salivating and burning. These attributes are positively associated with all green huajiao samples and a few red huajiao samples, specifically R4, R8, and R9. Astringency is located on the upper right of the *Y* axis and is positively correlated with red huajiao samples R5 to R7 and R11. In addition, bitter is located on the lower right of the *Y* axis and is positively correlated with red huajiao samples R1 to R3 and R10.

### 3.3 Hedonic responses

**Fig. 4:**
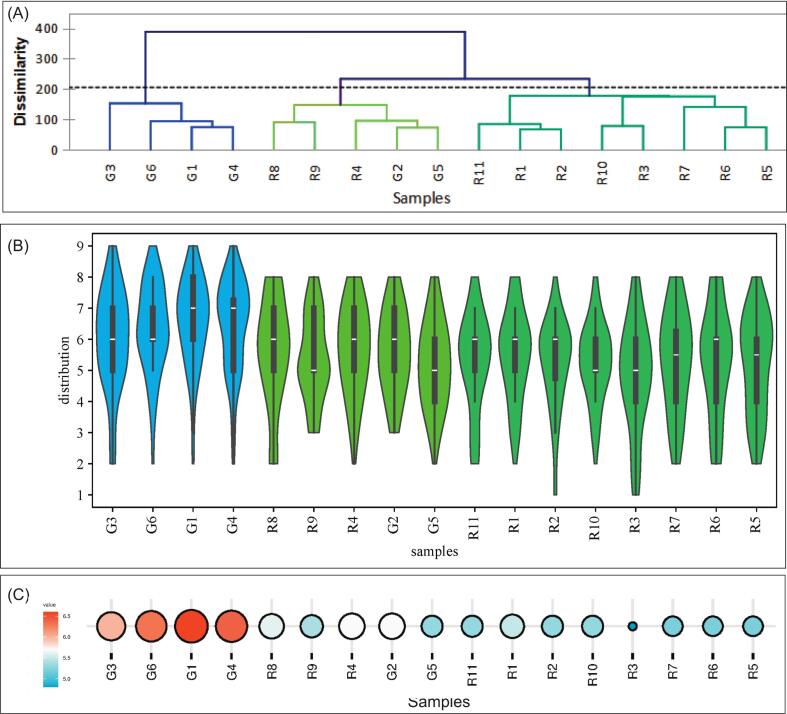
AHC analysis (A) and Violin plot (B) of overall liking scores for 17 test samples (blue color: cluster 1; light green color: cluster 2 A; dark green color: cluster 2B). Bubble Chart (C): Average Scores of All Samples. (For interpretation of the references to color in this figure legend, the reader is referred to the web version of this article.)

The distribution characteristics of overall liking scores for the pungency sensation of 17 test samples, rated on a 9-point scale by 60 participants are shown in Fig. 4. The AHC results indicated that the 17 huajiao samples were divided into two clusters based on Euclidean distance (Fig.4A). Significant differences were found among the 17 test samples in both the distribution of overall liking scores and mean liking scores (*p* < 0.05), as shown in Fig.4B and Fig. 4C. Four green huajiao samples (G3, G6, G1 and G4) had the highest liking scores, with mean liking scores ranging from 6.0 to 6.7, and were classified in Cluster 1 (blue color). These four samples had highest values for the upper quartile and the lower quartile and represented the samples with a higher preference. The other 13 samples were grouped into one cluster (Cluster 2, green color) and further divided into two subgroups. Cluster 2 A included five samples (R4, R8-R9, G2, G5) most of which had a higher upper quartile (7) and consistent box length. The mean liking scores for these samples ranged from 5.4 to 5.7. Cluster 2B comprised eight red huajiao samples, with mean liking scores for most samples between 4.8 and 5.3. Samples R1-R2 and R10-R11 had short box lengths and a higher lower quartile (about 5) compared with the other four samples (R3, R5-R7), whose mean liking scores ranged from 5.3 to 5.5. In addition, the clustering results of the liking scores showed a partial distribution pattern of varietal traits. Dahongpao huajiao (R1-R3) and Sichuan Nanjiao (R5-R7) were grouped into Cluster 2B, while Fujiao huajiao (R8-R9) was grouped into Cluster 2 A, and most of the green huajiao were grouped into Cluster 1.

### 3.4 Emotional responses

**Fig. 5:**
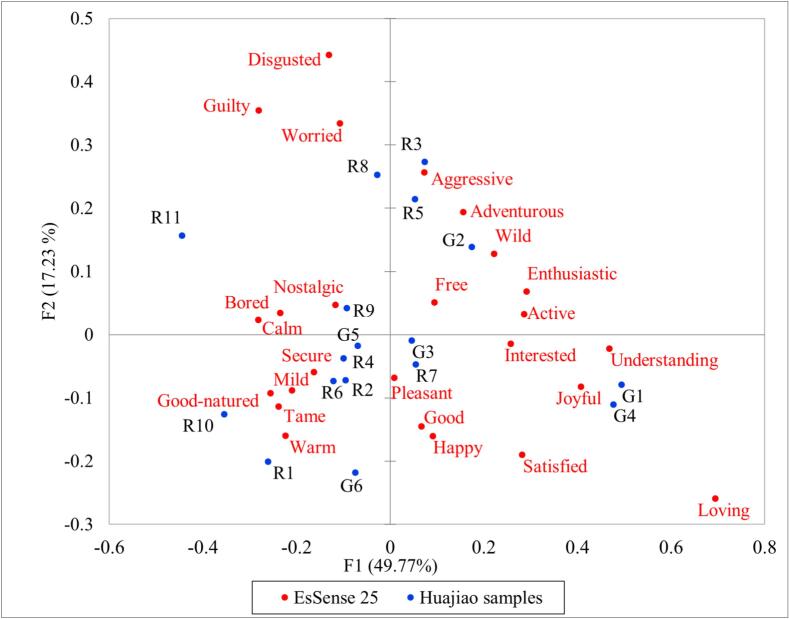
A bi-plot of the first two dimensions generated by Correspondence Analysis on the frequency of emotions for 17 huajiao samples (blue color for the huajiao samples red color for the emotions). (For interpretation of the references to color in this figure legend, the reader is referred to the web version of this article.)

The elicited emotional characteristics of 17 test samples were measured using the EsSense25 method. Cochran's Q-tests revealed significant differences in 17 out of 25 emotion terms among these samples, as shown in Supplementary Table 1 (*p* < 0.05). No significant differences were observed for the terms Free, Worried, Nostalgic, Pleasant, Good, Guilty, Happy and Interested (*p* > 0.05). Fig. 5 presents a two-dimensional representation of the first dimensions of the Correspondence Analysis (CA) based on the selection frequency of the 25 emotion terms for the test samples. The first two dimensions explained 67.0% of the variance, with the first dimension accounting for nearly 50%. Most green huajiao samples appeared on the right side of the y-axis, while most red huajiao samples were on the left. This distribution highlights the distinct emotional profiles between red and green huajiao variants in consumer perception. For example, G4 and G1 showed strong positive correlations with joyful, interested and understanding; G6 showed strong negative correlations with aggressive and adventurous while G2 showed strong positive correlations with these emotions. In contrast, most red huajiao samples demonstrated strong correlations (both positive and negative) with moderately subdued positive emotions, including secure, warm, tame, and mild. In addition, negative emotions such as worried, disgusted, and guilty showed weaker correlations with most test samples in the upper left areas, suggesting that the tested samples elicited predominantly positive or complex emotional responses rather than strongly negative emotions.

### 3.5 Correlations between the pungency and consumer preferences

**Fig. 6:**
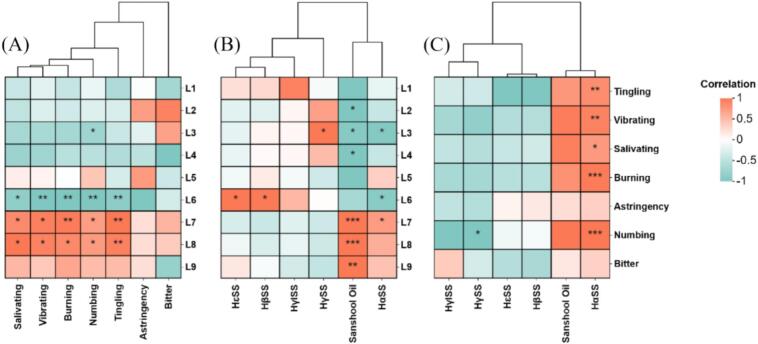
Correlations Cluster heat-map to Pungency perception VS liking scores (A), alkylamide content VS liking scores (B) and Pungency perception VS alkylamide content (C)for 17 huajiao samples. (The values represent Spearman correlation coefficients. * means *p* < 0.05; ** means *p* < 0.01; *** means *p* < 0.001).

Fig.6 shows a bivariate correlation clustering heatmap that visualizes the observed relationships among sensory pungency attributes, alkylamide compounds, and nine-point liking scores for 17 huajiao samples. In the heatmap, increasingly red colors indicate stronger positive correlations, while increasingly blue colors represent stronger negative correlations. Most pungency attributes were positively correlated with the segment of higher liking scores (7–8, like to like very much) except for astringency and bitter (Fig. 6A), although these relationships are correlative rather than causal. Six alkylamide compounds were divided into three groups (Fig. 6B). HαSS and sanshool oil were grouped together and were significantly positively associated with the segment of higher liking scores (7–9, like to like extremely). The second group included HβSS and HεSS, which tended to be positively associated with the medium liking score segment (5–6, neither like nor dislike to like slightly). The third group included HγSS and HγISS, which were mainly positively associated with the low liking score segment (1–4, dislike extremely to dislike slightly). These matrix relationships suggest that high liking scores are generally associated with higher contents of HαSS and sanshool oil, although causality cannot be inferred (Fig. 6B). In addition, the main pungency attributes were significantly positively associated with HαSS and sanshool oil (Fig. 6C).

**Fig. 7:**
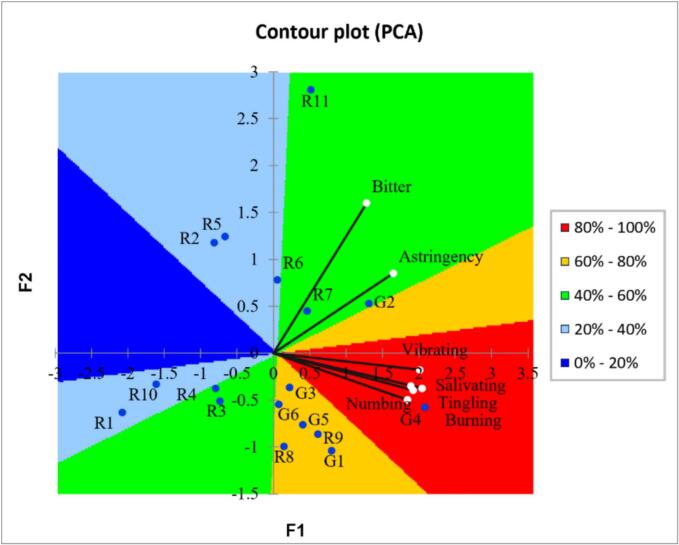
External preference map of liking score and sensory attribute for 17 huajiao samples.

An external preference map was also used to explore the correlations between consumer preferences and sensory attributes. Fig. 7 shows that the first two principal components, F1 (72.93%) and F2 (12.50%), together accounted for 85.43% of the total variance. Most green huajiao samples were positioned closer to higher liking scores and showed strong positive associations with sensory attributes of vibrating, salivating, burning, tingling and numbing, suggesting potential connections rather than direct causation. Most red huajiao samples were in the medium and medium-low liking areas and tended to be negatively associated with the sensory attributes of bitterness and astringency.

**Fig. 8:**
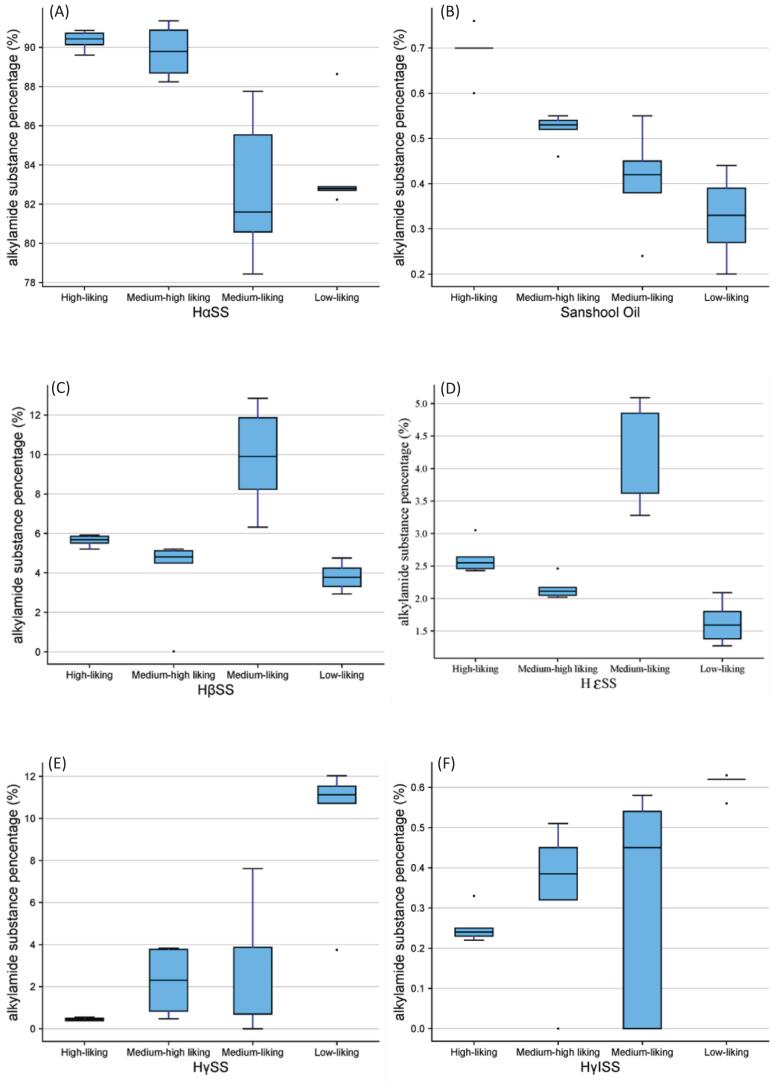
The concentration distribution of six alkylamide substances in different groups samples.

Additionally, the concentration distribution of six alkylamide percentage composition in different liking huajiao sample groups is shown in Fig. 8. The high-liking huajiao sample group (G1, G3, G4 and G6) had the highest concentration of HαSS and sanshool oil, with contents of 89.61% ∼ 90.86% and 0.60% ∼ 0.76%, respectively (Fig.8A and Fig.8B). The medium-liking huajiao sample group (R1, R2, R10 and R11) showed the highest concentration distribution of HβSS and HεSS, with contents of 6.32% ∼ 12.85% and 3.28% ∼ 5.09%, respectively (Fig.8C and Fig.8D). The low-liking samples (R3, R5-R7) had the highest concentration distribution of HγSS and HγISS, with contents of 3.75% ∼ 12.03% and 0.56% ∼ 0.63%, respectively (Fig.8E and Fig.8F).

### 3.6 Correlations between emotion response and consumer preference

**Fig. 9:**
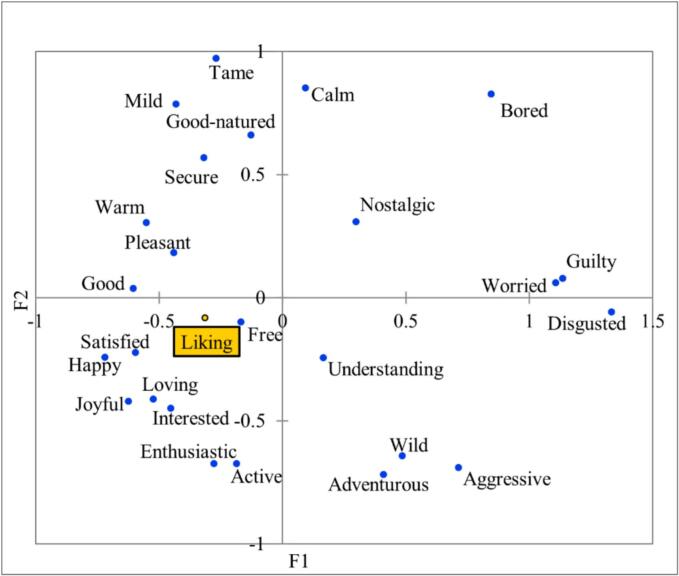
Principal Coordinate Analysis on the frequency of emotions and liking scores for 17 huajiao samples.

Principal Coordinate Analysis (PCoA) was conducted to explore the relationship between consumers' overall liking scores and the corresponding frequencies of elicited emotions for 17 huajiao samples (Fig. 9). The results indicated strong correlations between the liking scores and positive, pleasant emotions, including happy, satisfied, loving, joyful, interested, enthusiastic and active, which were located in the lower left area of the PCoA plot. In contrast, negative or less positive emotions, such as guilty, worried, disgusted, and bored, were located in the opposite area (upper right area), suggesting a strong negative correlation with the liking score.

**Fig. 10:**
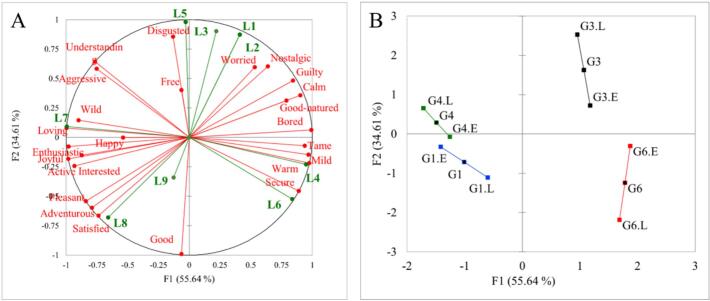
Bi-plot of the first two dimensions for emotions and liking scores (A) and coordinates plot of the projected points (B) for test samples in Cluster 1, generated by MFA.

Multiple Factor Analysis (MFA) was employed to examine the consistency between the overall liking scores and the elicited emotions of the high-liking huajiao sample group (G1, G3, G4 and G6). This analysis used the selected frequencies of 25 emotions and 9 point liking scores. The high-liking huajiao sample group had a high RV coefficient (0.754), indicating a close association between liking scores and elicited emotions. The first two dimensions of the MFA accounted for 90.25% of the variance, with 55.64% and 34.61% explained by the first and second dimensions, respectively (Fig. 10A). High liking scores (7–9) were in the lower right area and were positively associated with positive and pleasant emotions, while low liking scores (1–3) were in the opposite area and were associated with negative emotions. In addition, medium liking scores (4 and 6) appeared to be moderately correlated with the emotions warm, wild, secure and others. Fig. 10B shows that these four high-liking samples exhibited similar patterns in their original variable characteristics (emotion and liking score) and showed clustered coordinates in both variable dimensions, suggesting that these samples were associated with both high preferences and strong positive emotional characteristics.

## 4 Discussion

Significant variations in the concentration and composition of alkylamide compounds were found for dried huajiao of different origins (Zhu et al., 2025). Most red huajiao samples had higher total alkylamide contents than green huajiao samples, which is consistent with our results (Wang et al., 2025). Green huajiao samples had a higher HαSS percentage concentration than red huajiao samples, while red huajiao had a higher HγSS percentage concentration than green huajiao (Song, Zhao, et al., 2025). Chen et al. also demonstrated that the HγSS content in Sichuan nanjiao was significantly higher than in green huajiao and other red huajiao samples, while the HαSS content in green huajiao was higher than in red huajiao (Chen et al., 2022).

Previous research (Hu et al., 2026) has shown that green huajiao oil exhibits unique stimulative and functional characteristics. These profiles may contribute to the overall sensory experience and potentially influence consumer acceptance. Green huajiao samples were most preferred by consumers compared to the red huajiao sample with the highest total alkylamide content, suggesting that there may not be a simple linear correlation between consumer preference for the huajiao samples and total alkylamide content. In contrast, the percentage concentration of alkylamide compounds appeared to be more closely associated with consumer preferences for huajiao samples. Green huajiao samples (G1, G3, G4 and G6) had the highest percentage concentrations of HαSS and sanshool oil and were strongly preferred relative to samples containing only high levels of HαSS (G2 and G5), indicating a potential combined positive contribution of HαSS and sanshool oil to consumer preference. In a previous study, HαSS and sanshool oil were also identified as the key chemical compounds responsible for the characteristic numbing sensation in green huajiao, which showed discriminative power in classifying the varieties (Gao et al., 2024). HαSS exhibited a rapid onset of numbing stimulation (latency<15 s), imparted a distinctly strong spicy and numbing sensation intensity at a high level and was positively correlated with numbing sensation, vibrating sensation and salivating response (Feng et al., 2023). The mechanisms underlying alkylamide substances induced tingling are currently explained by two hypotheses: activation of TRPV1 and TRPA1 ion channels (Sugai et al., 2005) and inhibition of two-pore potassium channels (KCNK3, KCNK9, and KCNK18). It has been reported that TRPV1 and TRPA1 are molecular targets of HαSS and HαSS can suppress the two-pore potassium channels to induce numbness sensations, while HβSS lacks such bioactivity (Feng et al., 2022).

Nanjiao samples (R5-R7) exhibited higher total alkylamide content, HγSS concentration and pungency intensity, but lower consumer preference. HγSS also produces a strong numbing sensation because, like HαSS, it activates TRPV1/TRPA1 channels. In addition, at equimolar concentrations, HγSS elicited approximately a twofold increase in the duration of both tingling intensity (*p* < 0.01) and residual numbness (*p* < 0.05) compared to HαSS (Feng et al., 2023). This may partly explain why nanjiao samples (R5-R7) with low HαSS content still exhibited a strong pungency sensation, suggesting compensatory effects of high HγSS content. HγSS and HγISS differ from the fast and sharp stimulation of HαSS by producing a softer, slower and longer-lasting numbness. This observation may also help account for the broader distribution of consumer preference and lower mean liking scores, as longer duration and higher numbness intensity could reduce consumer preference. Therefore, huajiao samples with moderate pungency intensity, rapid perception rate and short duration may be more likely to be preferred by consumers.

The external preference mapping visualized the preferences for the huajiao samples in the attribute space. The results in Fig. 7 indicate that, with the exception of bitterness and astringency, most sensory attributes of pungency sensation showed positive correlations with consumer preference. This is consistent with previous reports that sensory attributes such as tingling, numbing, vibrating, burning and astringency are the most important sub-qualities contributing to the pungency sensation (Song, Xia, & Zhong, 2025). Bitterness is a negative factor influencing perceived flavor and consumer choice of huajiao (Huang et al., 2022). Huajiao samples that correlated strongly with bitterness and astringency generally had lower liking scores and fewer reported positive emotional responses. Current research on the flavor of huajiao mainly focuses on aroma and pungency sensation, while bitterness is rarely reported (Wang et al., 2025). Further research on the source of bitterness and its changes during storage is warranted for quality control and commercialization of huajiao.

PCoA plot results showed that high liking scores for pungency sensation samples tended to be were more strongly associated with emotions such as satisfied, happy, enthusiastic, loving, joyful, free, and interested. These associations are consistent with previous findings showing that food-evoked emotions are closely linked to consumer liking scores (Nervo et al., 2024; Rini et al., 2022). When researching emotional responses evoked by food, the two main dimensions of emotions, valence and arousal, must be taken into account. The valence dimension can be understood as an axis with pleasure and displeasure or attraction and aversion., at its ends. It is generally represented by the differentiation of terms as positive and negative. Arousal or engagement, is another key dimension of emotion that can be conceptualized as an axis with perceived activation and deactivation at its ends or as a feeling of high to low energy (Barrett, 2004). Most emotional responses evoked by food in a commercial context are positive (King & Meiselman, 2010). These emotions can be categorized as positive, more positive, negative, more negative, or with no clear classification. Other reports have confirmed this finding, showing positive correlations between the intensity of elicited positive emotions and liking scores for commercial dairy products and Australian Shiraz wines (Zhong et al., 2025). These results are consistent with our study. Negative emotions contrasted with the liking scores and showed strong correlations with the low liking scores. Medium-liking scores (4–6) were moderately correlated with more positive or neutral emotions (e.g., tame, wild, mild).

The association between sensory attributes and emotional responses provides an important explanatory dimension for understanding consumers' preferences for spicy foods. Sensations such as capsaicin-induced burning may contribute to high arousal and a range of emotional responses, from excitement to irritation, depending on individual tolerance (Simons et al., 2019). Nevertheless, the current analyses only describe correlation patterns and should be interpreted with caution regarding direct causal effects.

MFA allows for the simultaneous analysis of several variable tables. The advantage of this method is that it can analyze the relationships among observations, variables and tables (Nestrud et al., 2016). The variables in different tables can be of different types, but within each table, they must be of the same type (Schouteten et al., 2018). MFA method has been used to investigate the relationships between hedonic response, sensory perception in different participants and measurements and emotional response in many foods, including edible insect species, milk desserts, coffee beans, etc. (Ares et al., 2010; Nervo et al., 2024; Partida-sedas et al., 2019). These reports show that different types of food with different preferences are closely associated with emotional response or sensory perception. The RV coefficient is an important parameter of MFA and represents the relationships between the variables in two tables. The closer the relationships are, the closer the RV coefficient is to 1 (Worch, 2013). A strong relationship was reported between a 13-variable sensory profile and consumer liking ratings with an RV coefficient of 0.59 (Le & Husson, 2008). Most green huajiao samples (Cluster 1) had the highest mean liking scores (6.0–6.6) and showed strong associations between emotions and liking scores (RV = 0.754). This indicates that huajiao samples with high preference can also evoke the positive emotions.

Previous reports have shown strong correlations between age and flavor perception, with increasing age typically associated with decreased spice preferences (Guido et al., 2016). This study focused exclusively on a younger consumer group (*n* = 60, aged 18–28), and future research will include a wider age range and a larger sample size. In addition, a gel-like form was used instead of an aqueous solution for the test sample in this study to minimize the influence of product form on consumer preferences. The result of the paired difference test showed that the gel-like form did not alter the perception of pungency (*p* > 0.05). However, its potential impact on the relationship between chemical composition and consumer responses still requires further investigation in future studies. In daily culinary practice, huajiao is used as an auxiliary seasoning within complex food matrices (Schneider et al., 2014). Therefore, future research should use more authentic huajiao-flavored foods as test samples and include a wider range of pungency intensities and liking scores.

## 5 Conclusions

This study provides new insights into how the content and composition of alkylamide compounds in huajiao samples from different origins influence sensory perception and consumer preference. The results indicated significant differences in total alkylamide content and component profiles between green huajiao and red huajiao samples. These chemical differences were strongly associated with variations in consumer hedonic responses. Green huajiao samples elicited high levels of consumer preference, while red huajiao samples elicited a wider range of preferences. Higher percentages of HαSS and sanshool oil among alkylamide compounds were significantly correlated with increased consumer preference. This confirms that chemical composition jointly influenced consumer preference through both emotional response and sensory perception. In conclusion, future research should consider the different emotional responses to pungency sensation in various huajiao samples to develop a range of pungent foods that meet consumers' preferences and emotional expectations.

## CRediT authorship contribution statement

**Wen Qiao:** Writing – original draft, Methodology, Data curation. **Kui Zhong:** Software. **Bolin Shi:** Investigation, Formal analysis. **Houyin Wang:** Resources. **Wenxin Wang:** Formal analysis. **Yake Xiang:** Writing – review & editing. **Lihan Zhang:** Writing – review & editing. **Kai Su:** Validation, Resources, Conceptualization. **Na Wang:** Supervision. **Lei Zhao:** Project administration.

## Declaration of competing interest

The authors declare that they have no known competing financial interests or personal relationships that could have appeared to influence the work reported in this paper.

## Data Availability

Data will be made available on request.
