# Supplementary material for: From pungency perception to consumer preference: The driving role of alkylamide compounds in Zanthoxylum bungeanum
Source: Food Chem X. 2026 May 11;36:103972. doi: 10.1016/j.fochx.2026.103972 (PMC13196537; doi:10.1016/j.fochx.2026.103972)
Supplement: Supplementary file 1 — Supplementary material [file mmc1.docx]

**Supplementary Table 1** Frequency of selection and multiple pairwise comparisons of the emotional terms for the Seventeen different samples of huajiao

**Supplementary Table 2** Standard curve of hydroxy-ɛ/α/β/γ-sorcinin

**Supplementary Table 1**

|  | P-values | R6 | R2 | G1 | G4 | R11 | R10 | R3 | R4 | R5 | R9 | G6 | R7 | R8 | G2 | G3 | G5 | R1 |
| --- | --- | --- | --- | --- | --- | --- | --- | --- | --- | --- | --- | --- | --- | --- | --- | --- | --- | --- |
| Active*** | 0.000 | 0.233 (ab) | 0.267 (abc) | 0.517 (c) | 0.450 (bc) | 0.067 (a) | 0.150 (ab) | 0.233 (ab) | 0.233 (abc) | 0.350 (abc) | 0.250 (abc) | 0.217 (ab) | 0.317 (abc) | 0.283 (abc) | 0.367 (abc) | 0.250 (abc) | 0.267 (abc) | 0.183 (ab) |
| Loving*** | 0.000 | 0.100 (ab) | 0.050 (ab) | 0.283 (b) | 0.317 () | 0.017 (a) | 0.033 (a) | 0.067 (ab) | 0.083 (ab) | 0.033 (a) | 0.050 (ab) | 0.083 (ab) | 0.100 (ab) | 0 (a) | 0.133 (ab) | 0.150 (ab) | 0.033 (a) | 0.033 (a) |
| Mild* | 0.019 | 0.383 (ab) | 0.383 (ab) | 0.283 (ab) | 0.283 (ab) | 0.433 (ab) | 0.450 (ab) | 0.217 (a) | 0.417 (ab) | 0.267 (ab) | 0.300 (ab) | 0.367 (ab) | 0.333 (ab) | 0.300 (ab) | 0.367 (ab) | 0.333 (ab) | 0.383 (ab) | 0.567 (b) |
| Aggressive* | 0.018 | 0.233 (ab) | 0.133 (ab) | 0.250 (ab) | 0.250 (ab) | 0.217 (ab) | 0.117 (ab) | 0.350 (b) | 0.250 (ab) | 0.183 (ab) | 0.233 (ab) | 0.067 (a) | 0.233 (ab) | 0.283 (ab) | 0.283 (ab) | 0.217 (ab) | 0.183 (ab) | 0.133 (ab) |
| Bored* | 0.018 | 0.250 (a) | 0.283 (a) | 0.167 (a) | 0.167 (a) | 0.350 (a) | 0.400 (a) | 0.333 (a) | 0.200 (a) | 0.317 (a) | 0.400 (a) | 0.333 (a) | 0.300 (a) | 0.200 (a) | 0.167 (a) | 0.317 (a) | 0.317 (a) | 0.283 (a) |
| Calm** | 0.010 | 0.383 (a) | 0.367 (a) | 0.150 (a) | 0.217 (a) | 0.483 (a) | 0.433 (a) | 0.300 (a) | 0.433 (a) | 0.317 (a) | 0.367 (a) | 0.317 (a) | 0.300 (a) | 0.300 (a) | 0.233 (a) | 0.367 (a) | 0.317 (a) | 0.383 (a) |
| Wild*** | 0.000 | 0.167 (abc) | 0.233 (abc) | 0.333 (bc) | 0.450 (c) | 0.200 (abc) | 0.050 (a) | 0.300 (abc) | 0.167 (abc) | 0.133 (ab) | 0.167 (abc) | 0.200 (abc) | 0.233 (abc) | 0.383 (bc) | 0.283 (abc) | 0.250 (abc) | 0.233 (abc) | 0.167 (abc) |
| Disgusted* | 0.039 | 0.133 (a) | 0.067 (a) | 0.083 (a) | 0.067 (a) | 0.200 (a) | 0.083 (a) | 0.183 (a) | 0.067 (a) | 0.183 (a) | 0.117 (a) | 0.017 (a) | 0.067 (a) | 0.133 (a) | 0.133 (a) | 0.117 (a) | 0.067 (a) | 0.067 (a) |
| Understanding*** | 0.000 | 0.083 (ab) | 0.117 (ab) | 0.300 (b) | 0.350 () | 0.017 (a) | 0.050 (a) | 0.117 (ab) | 0.083 (ab) | 0.250 (ab) | 0.083 (ab) | 0.150 (ab) | 0.133 (ab) | 0.117 (ab) | 0.083 (ab) | 0.283 (ab) | 0.117 (ab) | 0.050 (a) |
| Warm* | 0.020 | 0.350 (a) | 0.167 (a) | 0.183 (a) | 0.150 (a) | 0.250 (a) | 0.317 (a) | 0.150 (a) | 0.250 (a) | 0.083 (a) | 0.233 (a) | 0.283 (a) | 0.233 (a) | 0.117 (a) | 0.183 (a) | 0.233 (a) | 0.217 (a) | 0.250 (a) |
| Enthusiastic  *** | 0.000 | 0.200 (ab) | 0.200 (ab) | 0.400 (b) | 0.383 (b) | 0.050 (a) | 0.117 (ab) | 0.333 (ab) | 0.267 (ab) | 0.250 (ab) | 0.200 (ab) | 0.200 (ab) | 0.333 (ab) | 0.183 (ab) | 0.383 (b) | 0.250 (ab) | 0.217 (ab) | 0.133 (ab) |
| Freens | 0.079 | 0.133 (a) | 0.233 (a) | 0.333 (a) | 0.267 (a) | 0.150 (a) | 0.133 (a) | 0.333 (a) | 0.200 (a) | 0.200 (a) | 0.200 (a) | 0.217 (a) | 0.150 (a) | 0.200 (a) | 0.250 (a) | 0.300 (a) | 0.300 (a) | 0.267 (a) |
| Tame*** | 0.001 | 0.433 (a) | 0.450 (a) | 0.250 (a) | 0.300 (a) | 0.483 (a) | 0.433 (a) | 0.300 (a) | 0.383 (a) | 0.183 (a) | 0.317 (a) | 0.450 (a) | 0.383 (a) | 0.300 (a) | 0.250 (a) | 0.383 (a) | 0.450 (a) | 0.550 (a) |
| Adventurousns | 0.103 | 0.167 (a) | 0.167 (a) | 0.283 (a) | 0.250 (a) | 0.150 (a) | 0.067 (a) | 0.283 (a) | 0.183 (a) | 0.233 (a) | 0.217 (a) | 0.167 (a) | 0.233 (a) | 0.250 (a) | 0.250 (a) | 0.133 (a) | 0.183 (a) | 0.117 (a) |
| Satisfied*** | 0.000 | 0.150 (ab) | 0.117 (ab) | 0.367 (b) | 0.317 (b) | 0.033 (a) | 0.117 (ab) | 0.133 (ab) | 0.133 (ab) | 0.117 (ab) | 0.150 (ab) | 0.217 (ab) | 0.183 (ab) | 0.117 (ab) | 0.167 (ab) | 0.150 (ab) | 0.183 (ab) | 0.200 (ab) |
| Worriedns | 0.153 | 0.083 (a) | 0.133 (a) | 0.117 (a) | 0.067 (a) | 0.217 (a) | 0.133 (a) | 0.167 (a) | 0.133 (a) | 0.183 (a) | 0.083 (a) | 0.083 (a) | 0.117 (a) | 0.183 (a) | 0.183 (a) | 0.167 (a) | 0.117 (a) | 0.033 (a) |
| Nostalgicns | 0.093 | 0.183 (a) | 0.150 (a) | 0.117 (a) | 0.067 (a) | 0.083 (a) | 0.233 (a) | 0.133 (a) | 0.083 (a) | 0.217 (a) | 0.100 (a) | 0.100 (a) | 0.183 (a) | 0.100 (a) | 0.133 (a) | 0.200 (a) | 0.133 (a) | 0.100 (a) |
| Pleasantns | 0.066 | 0.217 (a) | 0.217 (a) | 0.350 (a) | 0.333 (a) | 0.217 (a) | 0.333 (a) | 0.150 (a) | 0.183 (a) | 0.267 (a) | 0.233 (a) | 0.233 (a) | 0.167 (a) | 0.217 (a) | 0.167 (a) | 0.217 (a) | 0.100 (a) | 0.250 (a) |
| Secure* | 0.053 | 0.350 (a) | 0.450 (a) | 0.317 (a) | 0.233 (a) | 0.317 (a) | 0.467 (a) | 0.283 (a) | 0.433 (a) | 0.300 (a) | 0.283 (a) | 0.500 (a) | 0.383 (a) | 0.400 (a) | 0.300 (a) | 0.333 (a) | 0.433 (a) | 0.383 (a) |
| Goodns | 0.204 | 0.183 (a) | 0.250 (a) | 0.317 (a) | 0.267 (a) | 0.100 (a) | 0.217 (a) | 0.167 (a) | 0.200 (a) | 0.117 (a) | 0.167 (a) | 0.267 (a) | 0.233 (a) | 0.167 (a) | 0.217 (a) | 0.200 (a) | 0.283 (a) | 0.217 (a) |
| Good-natured*** | 0.001 | 0.367 (ab) | 0.383 (ab) | 0.133 (a) | 0.300 (ab) | 0.417 (ab) | 0.467 (b) | 0.200 (ab) | 0.400 (ab) | 0.283 (ab) | 0.317 (ab) | 0.367 (ab) | 0.383 (ab) | 0.250 (ab) | 0.200 (ab) | 0.383 (ab) | 0.233 (ab) | 0.400 (ab) |
| Guilty* | 0.032 | 0.033 (a) | 0.033 (a) | 0.017 (a) | 0.033 (a) | 0.100 (a) | 0.150 (a) | 0.067 (a) | 0.050 (a) | 0.150 (a) | 0.100 (a) | 0.050 (a) | 0.067 (a) | 0.150 (a) | 0.067 (a) | 0.067 (a) | 0.083 (a) | 0.033 (a) |
| Happyns | 0.058 | 0.100 (a) | 0.117 (a) | 0.150 (a) | 0.200 (a) | 0.033 (a) | 0.183 (a) | 0.050 (a) | 0.083 (a) | 0.150 (a) | 0.150 (a) | 0.133 (a) | 0.200 (a) | 0.083 (a) | 0.067 (a) | 0.133 (a) | 0.083 (a) | 0.083 (a) |
| Interested** | 0.011 | 0.117 (a) | 0.117 (a) | 0.333 (a) | 0.250 (a) | 0.100 (a) | 0.133 (a) | 0.150 (a) | 0.200 (a) | 0.150 (a) | 0.100 (a) | 0.100 (a) | 0.150 (a) | 0.100 (a) | 0.150 (a) | 0.150 (a) | 0.117 (a) | 0.083 (a) |
| Joyful*** | 0.000 | 0.150 (abc) | 0.167 (abc) | 0.367 (bc) | 0.383 (c) | 0.033 (a) | 0.100 (abc) | 0.133 (abc) | 0.100 (abc) | 0.183 (abc) | 0.150 (abc) | 0.167 (abc) | 0.250 (abc) | 0.117 (abc) | 0.183 (abc) | 0.183 (abc) | 0.133 (abc) | 0.067 (ab) |
| *** Indicates significant differences between samples according to Cochran’s Q test at p < 0.0001.  ** Indicates significant differences between samples according to Cochran’s Q test at p < 0.01.  * Indicates significant differences between samples according to Cochran’s Q test at p < 0.05.  ns Indicates no significant differences between samples according to Cochran’s Q test (p < 0.05).  1 Post hoc multiple pairwise comparison were performed using McNemar’s test with Bonferroni alpha adjustment.  The different letters (a, b, c) denote significant significance differences within the attribute at p < 0.05. | | | | | | | | | | | | | | | | | | |

**Supplementary Table 2**

| Substance. | Regression equation | Concentration range(μg/mL) | Correlation Coefficient R² |
| --- | --- | --- | --- |
| hydroxy-ɛ- sorcinin | y =89.15x + 7.88 | 0.50-5.00 | 0.9993 |
| hydroxy-α- sorcinin | y = 81.96x + 649.99 | 20.00-200.00 | 0.9992 |
| hydroxy-β- sorcinin | y = 132.85x + 24.9 | 1.00-10.00 | 0.9998 |
| hydroxy-γ- sorcinin | y = 134.46x + 28.60 | 0.25-15.00 | 0.9995 |
